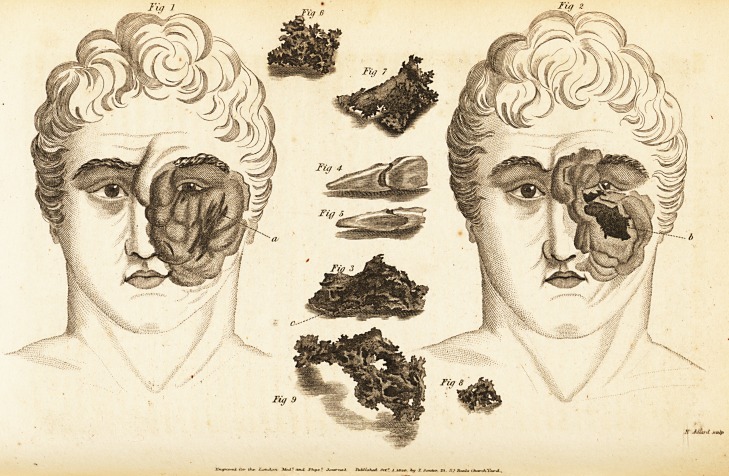# A Series of Cases, Exhibiting the Effects of Pressure in Cancerous and Other Diseases

**Published:** 1820-10

**Authors:** Samuel Young

**Affiliations:** Surgeon to the Cancer Institution, Gerard-street, Soho; Member of the Royal College of Surgeons, and of the London Medical and Chirurgical Society, &c.


THE LONDON

### Medical and Physical Journal.

4 OF VOL. XLIV.] OCTOBER, 1820. [NO. 260.

"For many fortunate discoveries in medicine, and for the detection of numerous "errors, the world is indebted to the rapid circulation of Monthly Journals; "and there never existed any work to which the Faculty in EUROPE and "AMERICA were under deeper obligations than to the Medical and Physical "Journal of London, now forming a long, but an invaluable, series." RUSH.

### Original Communications, Select Observations, etc.

FOR THE LONDON MEDICAL AND PHYSICAL JOURNAL.

### *A Series of Cases, exhibiting the Effects of Pressure in Cancerous and other Diseases.* By SAMUEL YOUNG, ESQ. Surgeon to the Cancer Institution, Gerard-street, Soho; Member of the Royal College of Surgeons, and of the London Medical and Chirurgical Society, &c.

IN presenting further evidence of the extraordinary and important effects produced by pressure in cancerous and other complaints, I should have confined myself, without comment or remark, to a simple statement of facts, had not the acts of prejudiced, presumptuous, and proscriptive power, of sordid and vindictive vanity, called for a still continued notice of that persecution,* so perseveringly, though privately, exerted by certain individuals of the profession, to crush the practice altogether, by a systematic stifling of evidence in its favour, by gross misrepresentations of facts and cases; indeed, by the fabrication of statements in their formation wholly untrue, for the sole purpose of prejudicing the minds of patients, as well as practitioners themselves, in order to deter them from venturing on the practice.†

* See the *Further Reports of Cancerous Cases, &c.* published by the author in 1818, where the base and very criminal conduct of certain individuals is alluded to, and exposed.

† The following may serve as a specimen, among the many instances of professional misrepresentation. A medical gentleman, of considerable talent and great respectability, whose lady I now attend for a confirmed scirrhus of the whole left breast, was assured, but a short time since, by an hospital surgeon and a public lecturer, when consulting him on the case, and as touching the practice of pressure, "that all my patients, if they recovered from the disease, ultimately died of *rheumatism!*" An assertion in all its parts entirely gratuitous, and evidently fabricated solely to deter and depreciate; as the gentleman, though considerably prejudiced by it in the first instance, upon further enquiry, to his full conviction, found to be the case. It may be proper to add, that the surgeon alluded to is since dead. The above mentioned case is now subsiding, under pressure, in the most satisfactory and promising manner.

Had it not been for such a combination of undue influence, I repeat, I should have contented myself by a simple detail of facts, without any attendant remarks, which now, however, become requisite, to warn the medical public in general against the influence of such prejudiced representations, which are constantly being exercised against the practice, to render it unjustly odious and obsolete.

How long such influence is solely to direct the judgment of the public, and issue out its limits for other men's comprehensions, would be a difficult speculation to decide. When the slave is released for ever from the galley, and folly stripped of her grave formalities, then, perhaps, a medical man may contemplate nature as she is, free from the fetters of the schools and the dogmas of the profession; and, without 'pains and penalties,' venture to promulgate truths as they suggest themselves to his mind, from the observation of facts, confirmed and consolidated by the repetitions of experience.

In giving to the world, however, the discovery of the new method of treatment by pressure, the principle of which involves so extensive and important a circle of practice in so many various diseases, it was offered, as the present cases I am about to relate now are, regardless of every other consideration but that of truth; in the hope and trust, that there is quite sufficient intellect of comprehension, and ability and independant honesty, whatever may be the confederated efforts of a few prejudiced and mercenary individuals to the contrary, to establish, enlarge, and perpetuate the practice, for the benefit of mankind.

Individual notice and altercation, as far as it has been permitted me, I have always particularly avoided; and on the present occasion I drop, as much as possible, all personal allusions, contenting myself generally to apprize the medical public of the existence of such undue influence, that they may not be prejudiced by misrepresentations, nor submit in a succumbing spirit to the direction of others, but fairly and manfully examine into facts, and judge for themselves.

To suppress truth by private cabal and secret influence, and meanly to raise a gossip-tale, argues any thing but a right and honourable application of talent and power. Such conduct, however successful for a while, only evinces the folly of a transient and compromised triumph, at the expense of character, and the indelible stain of infamy which ever attaches to the memory of talent prostituted and power debased.

The following series of cases is faithfully submitted, for the information and consideration of the profession at large. They show, in common, that the evidences of truth, however baffled or retarded, can never ultimately be stifled or destroyed.

H Adlard sculp

Engraved for the London Med.l and Phys.l Journal  Published Oct.r 1 1820, by J. Souter, 73, St Pauls Church Yard.

Case I.—*The Case of Alexander Johnston, (with Engravings).*

The following very extraordinary and interesting case is thus put down in the minute-book of the Cancer Institution, Gerard-street. The history of the case is taken chiefly from the patient, as he himself stated it.

Alexander Johnston, age 53; Princes-street; June 5, 1819.—A cancerous tumor and sore of considerable elevation, occupying the entire of the upper part of the face on the left side. Extending from the os nasi and the inner canthus of the eye, across and over the os mali, and down the whole course of the nose, lip, and cheek connected with the upper maxillary bone. Firmly and immovably fixed to the bones throughout the whole of its origin. The covering integument deeply diseased and discoloured in many parts, with enlarged blood-vessels. Tumor has also extended itself over the inner angle of the opposite eye, and has likewise made progress just above the nose on the os frontis, where the protuberance is very evident.

June 4th, examined by Mr. Pearson, the consulting-surgeon to the Institution: this day (June 5), by Mr. Babington, the assistant-surgeon.

The patient states as follows. About two years since, first had a flowing of the tears of the left eye, attended by inflammation of the conjunctiva. Some three months after, had a severe cough. At this time a swelling came on, just over the cheek-bone, which became red, and variously discoloured; and which (the patient thinks) was a good deal aggravated by the cough. The swelling increased, came to a head, and broke, discharging a small quantity of thick white matter, but leaving a considerable hard substance, or core, behind. In this state the disease was poulticed. The patient then applied to the Westminster Infirmary for the Diseases of the Eye, in Mary-la-bonne-street, Golden-square; and the operation for fistula lachrymalis was performed by Mr. Guthrie. The patient wore a bougie in the canal for a fortnight, and then returned to his work; his trade a baker. From his occupation, the bougie could not be retained; the parts healed up, and all former apparent disease disappeared.

In about two months after, (the patient being in the habit of examining the part,) a very small, but hard, body was perceptible, just upon the upper part where the operation had been performed. This, from the size of a pin's head, soon increased to that of a pea, and then made rapid progress down the side of the nose and across the face, till the disease arrived at its present extent and bulk.

During its course, the disease always seemed to spring from the covering of the bone. This state of things occupied the space of six months. About this period he consulted Sir W. Adams, who sent him to Mr. A. Cooper; by whose advice leeches were applied to the tumor three times a-week. These were used for a fortnight, till their bites degenerated into a sore; its character that of a fungus.

In this state poultices were also used, and other medicines taken. The disease increased, till the patient says "he had no breath whatever through his nose." This part now is quite distorted, and the left nostril dragged outwards by the tumor towards the ear, as if tearing from it. There is also evident tumor to be felt through the whole upper lip immediately under the nostril of that side; and through the skin enlarged dark-coloured vessels are to be seen, forming an irregular network, in connection with those of a still more diseased state covering the nose and upper parts of this disease. Mr. Cooper's advice was, that the patient should return to his native air, (Scotland,) "as his disease was never got the better of."

The disease still advanced, making progress over the nose to the opposite side. Mr. Henry Earle was then seen in January of this year (1819), who sent the patient to Mr. Wardrop: he was then returned to Mr. Earle, and a drawing was taken of the disease. The patient says "he sat to an artist for his likeness, and had two or three sittings."

From this the patient went to the Westminster General Dispensary, Gerard-street, under the care of Mr. Harding. Here a considerable quantity of fungus was removed by the knife. This part now is on a level, or rather below, the surface of the tumor, and is situated at the outer side, as at (*a*), Plate I.* The patient then came to this Institution.

The patient's health is much disturbed, and he suffers considerable pain from the disease. He was put on an alterative plan of small doses of calomel and antimony about twice a-week; with the decoction of dandelion-root, a pint daily.

*June* 5, 1819.—First application of pressure with lint, firm linen compresses, and a double-headed roller.

6.—Removal. Evident general improvement. Pressure re-applied. No pain from the application, except the turns made by the roller about the ears, and which, in the re-application, were particularly avoided.

8.—Decided improvement. The large bulk of the tumor at the lower and outward side feels, under the integument, in a broken or divided state. The tumor over the nose and at the inner angle of the eye, the patient states himself as "evidently

* This Plate affords a general appearance of the disease when the patient first applied to this Institution.

decreased." After the pressure is applied and become settled, the patient also states that the whole of the diseased parts is much easier than formerly; and to his feelings has an agreeable warmth.

10.—Improvement continues. The sides, as well as bulk, of the tumor much diminished. Some sloughy appearance on the surface of the sore. The parts easy. Pressure continued, with increase. Haustus purg.

12.—Removal. The disease evidently diminished. Some superficial slough of the sore. The last three days, a considerable purulent discharge from both nostrils. The patient states that he feels his nose much more open, and breathes more easily. The parts have remained free from pain. Pressure continued.

20.—Removals every second or third day. Sloughing of the tumor increased. Discharge offensive. Parts easy. Very active pressure kept up.

*July* 10.—The sloughing has greatly extended. The tumor from the side of the nose to the outer part of the face, is excavating fast. The discharge from the tumor and the nostrils excessive, and highly offensive. No pain; although considerable irritation of the surrounding integument exists from the discharge.

30.—Sloughing continues. Masses of the tumor have come away. A portion of the orbicular bone under the eye at the outer side, exposed. The parts have remained free from pain. The pressure actively kept up.

*August* 20.—The orbicular portion of the bone largely exposed, of a dark colour, and becoming loose. Plate No. II. represents the general appearance of the disease about this period; (*b*) is the diseased portion of bone alluded to.

The patient states, that a few days back, whilst walking down Wardour-street, one of his front teeth fell out, and was lost. A portion also of the alveolar process came away, and which he brought with him.

The tumor of the upper lip has subsided, and the great stretching of the left nostril has been removed for some time.

Removals of the applications have been made every second or third day, and the pressure most actively employed. A tea-spoonful of the bark and sarsaparilla powders, in equal parts, has been given three times a-day.

*Sept.* 20.—A few days after the last report, the loose portion of the orbicular bone was removed by the forceps. (See fig. III. *c*.) From the great cavity produced, and want of support inwardly, the eye itself sunk, or rather fell, considerably towards the nose; the bone of which was now exposed with its natural covering, and its edge distinctly seen suspended over the general hollow. Two more of the upper incisors came away, and were preserved. (Figs. 4 and 5.) Plates of bone forming the outer wall of the maxillary sinus then began to exfoliate, exposing that cavity. (Figures 6, 7, 8, represent some of these portions.) Then a considerable solid portion of the maxillary bone itself was removed by the forceps upwards within the cavity, the covering integument of that part of the face remaining entire.

In the elevation of this portion of bone, two of the lateral incisors (or one incisor with the canine) were carried up with it; but, the gum still adhering to them and their portions of the alveolar, they were disengaged from the general mass by a slight twist of the main portion of the bone, and left adhering, though considerably raised above their former situation, and almost buried in the gum. Afterwards, when looking down into the maxillary sinus, the fangs or ends of these teeth could be distinctly seen, and were moved upon the slightest touch of the patient's tongue on the teeth in the mouth.

At the alveolar end of the removed portion of the maxillary bone, the remains of two sockets were visible. (Fig. 9, represents this portion of the maxillary bone.) Though exactly like it as it now is, yet the bone has become much more fossile by keeping, and at the time the sketch was taken, than it was upon its first removal. Fig. 9 . . . . 9, were the points where the alveolar processes could be traced, but which, from the lightness and change in the bone, are now very indistinct.

The discharge generally has been very copious, and so offensive at times, as to be almost insupportable. The tumor above the nose has either sloughed away, or detached portions removed by the scissars and forceps two inches from under the integument; the process of separation being by suppuration or abscess of the intervening cellular structure, separating it from, and leaving, the integument entire. For a very interesting process under the treatment by pressure for the removal of morbid parts, as already instanced in several cases, (see *Further Reports*, &c. published in 1818.)

The disease seems very generally to be cleared away. The immense cavity formed by the opening of the maxillary sinus, &c. has a most healthy appearance; in no one part is there a disposition to throw up fungus, and the discharge, generally, is a very good pus.

During the author's lectures last autumn, he was favoured by the constant attendance of Mr. Haden, of Sloane-street; of Dr. Armstrong, physician to the Fever Institution; of Mr. Le Mann, Orchard-street, Portman-square; and other professional gentlemen, to whom this case is familiar; and he feels assured, by the expressions they frequently used at the time, that he meets the feelings of those gentlemen, when he says, that no description can at all be adequate to the realities of the case, to the extraordinary features of its progress, or the very curious, interesting, and highly gratifying results, effected under the course of its treatment. The detail given, therefore, is only an attempt at a simple, though faithful, outline of this case.

Along the ridge of the nose some skin has begun to form.

*October* 30.—The cavity has very much contracted; the aperture, which formerly would admit a moderate-sized fist, will scarcely allow the first joint of the thumb to pass. The nose-sore, as well as all the surrounding parts, are forming firm healthy cuticle up to the very margin of the opening. The great loss of substance of the maxillary bone, &c. is so filled up as not to be perceptible to the eye, and scarcely to the touch, on examination. The enlarged diseased blood-vessels have disappeared. Very good pus, with the natural mucous discharge of the membranes of the parts, coat the pledgets of lint which fill up the cavity. And here it may be noted, that nothing but fine flocculent lint, and linen compress with the roller, has been used throughout in the treatment of this disease. Lint has always filled the cavity of the sinus, but with considerably more firmness at the early stages than latterly. No disposition to throw up fungus has been evinced; nor any disease whatever among the spongy structures of the nose-bones, &c. where so much *a priori* might have been dreaded. However successful the course of the treatment may be considered, yet all has not gone on so smoothly as not to present some difficulties. Parts of the disease have been very obstinate and irritable, connected with general constitutional disturbance, and evidently arising or aggravated by the state of the weather.

The greatest delicacy and management has been required in the local treatment, connected with the manner of applying the compresses at different periods. Such diseases are not to be conquered by what is called binding a piece of linen on *hard* or *tight*. *Mind* and *a good intention* must be carried along with the practice, for success to follow: a good intention, an *endeavour* to succeed, by contemplating the various circumstances of the case, and applying and varying the remedies with zeal and ability. Barbarous and unscientific bandaging, so far from succeeding, must aggravate all the circumstances of the disease to which it is applied. This subject, and the mode of application, is very fully treated in the *Further Reports of Cancerous Sores*, &c.* already alluded to; but too much importance cannot be attached to the subject.

* Published by Ridgway and Cox.

It has been omitted to observe, in the first instance, that the disease in this case was making progress into the mouth, and a spongy substance had penetrated through the bony palate about its centre. This has subsided; and the teeth, formerly mentioned as being elevated, and left attached to the gum on the removal of the maxillary portion of bone, have now become nearly firm, by the adhesion and growth of parts around them.

During the course of treatment, for the objects of science and utility, and out of individual consideration, the patient has been sent several times to Mr. Guthrie for inspection; and who on these occasions exhibited the case to his pupils and class, at the Infirmary in Mary-la-bonne Street.

With similar motives, the patient was also sent to the Westminster Dispensary, for Mr. Harding's inspection.

*March* 1820.—Since the last four months the cure has been drawing to a successful close, though variously interrupted and aggravated by the severity of the season, coupled to the very reduced circumstances of the patient, which have exposed him to great hardships and privations, inducing catarrhal attacks and fever. Besides which, the patient has been frequently induced, from his greatly improved state, considering the disease as gone, to relinquish altogether any application of pressure, and return to his occupation as baker, than which a more injurious calling could not be pursued: exposed not only to heat and sudden transitions, but also to the fatigue and consequent irritations of night-work. Under these circumstances, a fulness and sense of tumor returned at the inner angle of the eye, but which was wholly removed upon the re-application of the pressure. There was also some thickening to be felt over the cheekbone; and, during great catarrhal irritation, some glands enlarged at the angle of the jaw under the left ear: but these symptoms, however, have nearly subsided, by a little care and management.

All the former disease has skinned over, and good sound cuticle has formed within the very margins of the cavity left just under the inner angle of the eye; the under lid of which, by the cicatrization within the upper margin of this little cavity, is now drawn somewhat fixedly downwards, and the inner surface of the lid, in a slight degree, turned in consequence outward. From the loss of bone, &c. at this part, the muscles of the eye on this side would also appear to be impaired in their function, or injured in their insertions. There is evidently not so much freedom of motion of the ball, and it is drawn outward, and somewhat upward, giving to the eye a slight cast in that direction, and a fixed appearance. The patient himself, however, does not complain of any impaired vision, or other impediment.

The remaining aperture into the sinus is now so surrounded and filled up as scarcely to admit the point of the little finger, and in appearance, as indeed in fact, it is just similar to a third nostril, opening obliquely under the bones of the nose, instead of the bottom of the cartilage, and leading into the maxillary sinus, which, of course, must be considered under any circumstances as an open cavity, having naturally an external communication.

At this time the patient is induced to return to Scotland, in consequence of a conveyance on ship-board being offered him by his brother. The state of the weather at this inclement season is strongly urged against this resolution; but the man seems determined, at all hazards, to escape from his present state of penury and want. If determined to go, he is particularly requested to call upon Mr. Pearson for his inspection, who has seen the case from time to time.

In consequence of the ship sailing sooner than he calculated upon, Johnston is gone to Scotland, without either calling on Mr. Pearson, or coming to me for his last instructions. He has left word, however, that he will report the state of his case at some future period.

*August* 11, 1820.—The patient writes from Glasgow, saying, "that he is about entering into business." He also states, that after his arrival "the disease again came on between the opening and the ear to a frightful sight, and then broke, and discharged a kind of bagful of matter; and since that the disease declined:" "that his friends got several medical gentlemen to see him, who said they could do nothing more; but recommended him to pursue the plan of pressure, which was the only way to make a complete cure; and this he did." The patient then adds, "I will be enabled to do something for my bread; and I do firmly believe, had it not been for your taking me under your care, and paying such unremitted attention to me, I not only should have lost my eyes, but life also."

The patient, in his gratitude, then offers his best wishes for me, (which I state, to show his sense of the eminent benefits he has derived from the treatment, and consequently fair evidence conjunctively with other corroboration;) and this, as far as I know, is a faithful detail of this interesting and extraordinary case.

The patient also states, that, should he come to London, he will make a point of calling.

[*To be continued.*]

## Figures and Tables

**Fig 1 Fig 6 Fig 7 Fig 4 Fig 5 Fig 3 Fig 9 Fig 8 Fig 2 f1:**